# Survey and analysis of the quantitative methods used in electricity research on GCC countries: 1983–2018

**DOI:** 10.1016/j.heliyon.2019.e02634

**Published:** 2019-10-15

**Authors:** Mohammed A. AlKhars

**Affiliations:** KFUPM Business School, Department of Information Systems and Operations Management, KFUPM, Box 5076, Dhahran 31261, Saudi Arabia

**Keywords:** Energy, Energy economics, Renewable energy resources, Energy sustainability, Energy conservation, Urban energy consumption, Electricity, Demand and supply, Literature review, GCC, Analysis tools, Renewable energy

## Abstract

This study provides a systematic analysis of research on the electricity sector in Gulf Cooperation Council (GCC) countries in the period 1983–2018. GCC countries have experienced tremendous economic growth in the past few decades. This was accompanied by a corresponding increase in electricity consumption. Therefore, a thorough review is needed to understand the research conducted on the electricity sector in GCC countries. This study reviewed articles published in five well-known energy journals: Applied Energy, Energy, Energy Economics, Energy Policy, and Renewable and Sustainable Energy Reviews. The articles were classified into seven categories based on the analysis tools implemented in the papers: 1. Simulation tools, 2. Scenarios tools, 3. Equilibrium tools, 4. Top-down tools, 5. Bottom-up tools, 6. Operations optimization tools, and 7. Investment optimization tools. This study also provides an overview of the research, including the increase in publications over time, an authorship analysis, a keywords analysis, and an analysis of the length of the publications.

## 1 Introduction

Gulf Cooperation Council (GCC) countries have experienced tremendous economic growth during the last four decades. This economic growth was accompanied by corresponding growth in electricity demand and supply. The average growth rate of electricity consumption per capita for GCC countries in the period 1971–2012 is 6.2% (Osman et al., 2016). Because of this marked increase in electricity demand and the expensive nature of investment in the electricity sector, several academic research papers have been published that address various aspects of demand and supply in GCC countries. This study aims to provide a synthesis of the research published in five well-known energy journals. These journals are *Applied Energy, Energy, Energy Economics, Energy Policy,* and *Renewable and Sustainable Energy Reviews (RSER).* To the best of the author's knowledge, this is the first study to provide a bibliographic analysis of current electricity supply and demand research in GCC countries. The author hopes that this literature review will provide researchers with a comprehensive understanding of electricity supply and demand issues in GCC countries. This review may also help these researchers to investigate important areas not yet explored. The author has attempted to include all publications pertaining to the supply and demand of electricity in GCC countries. However, there is no guarantee that all papers are included or identified. If any paper was published in the five aforementioned journals and not included in this research, please feel free to contact the author.

## 2 Main text

### 2.1 Methodology

Literature reviews on published research in a specific scientific domain is critical for gaining a deeper understanding of the relevant issues and problems of the research area. For example, Emrouznejad and Yang (2018) provided a comprehensive list of published articles that used Data Envelopment Analysis (DEA) as an analysis tool. In the energy domain, Pfenninger et al. (2014) reviewed energy systems modeling for twenty-first century energy challenges. They grouped the models into four categories: energy systems optimization models, energy systems simulation models, power systems and electricity market models, and qualitative and mixed-methods scenarios. Jebaraj and Iniyan (2006) also provided a review of energy models. They discussed various energy models such as energy planning models, energy supply–demand models, forecasting models, renewable energy models, emission reduction models, optimization models, and models based on neural network and fuzzy theory. Additionally, Payne (2010) reviewed literature specifically related to the causal relationship between electricity consumption and economic growth. Finally, Connolly et al. (2010) reviewed the computer tools used to analyze the integration of renewable energy. They classified these tools into seven categories: 1. Simulation tools, 2. Scenarios tools, 3. Equilibrium tools, 4. Top-down tools, 5. Bottom-up tools, 6. Operations optimization tools, and 7. Investment optimization tools. This review followed the work of Connolly et al. and used this method to classify the research on GCC electricity systems. The review will assist both researchers and decision makers by increasing their awareness of existing research in the electricity sector in GCC countries and to focus future research on important areas that have received less attention in the past years.

The literature review implemented in this paper focuses on studies published in top tier journals on energy research. The five journals selected for this purpose are *Applied Energy, Energy, Energy Economics, Energy Policy,* and *RSER.* These journals were selected to ensure that high quality papers are considered in this analysis of the electricity sector in GCC countries. All of these journals are published by Elsevier and are considered to be Q1 (a journal ranking based on quartile scores and impact factor) journals. Table 1 provides the impact indicators of the five journals using the H-index and the Scimago Journal Rank (SJR).

**Table 1:** List of journals used for the literature review.

Journal Name	Impact Indicators		
	Quartile	5-year Impact Factor	SJR
Applied Energy	Q1	7.888	3.162
Energy	Q1	5.582	1.990
Energy Economics	Q1	4.963	1.916
Energy Policy	Q1	5.038	1.994
RSER	Q1	10.093	3.036

The author used the following seven keywords to search for relevant articles: Saudi Arabia, United Arab Emirates, Oman, Bahrain, Qatar, Kuwait, and GCC. Only articles related to the supply or demand of electricity were used in the analysis. Other articles related to other fields—such as analysis of weather data, materials, or the production of oil and gas—were excluded from the analysis. This process was conducted for each of the five journals, and a total of 206 articles were found. The detailed distribution of this number among the five journals and among the different GCC countries is shown in Table 2.

**Table 2:** Numbers of articles published by journals and the countries studied.

Journals/Country	Bahrain	GCC	Kuwait	Oman	Qatar	Saudi Arabia	UAE	Total	%
Applied Energy	7	2	8	6	0	16	13	52	25%
Energy	3	2	8	6	1	13	4	37	18%
Energy Economics	0	2	3	0	0	2	0	7	3%
Energy Policy	1	8	4	0	0	16	5	34	17%
RSER	2	12	5	13	2	27	15	76	37%
Total	13	26	28	25	3	74	37	206	100%
%	6%	13%	14%	12%	1%	36%	18%	100%	

RSER published 76 articles, the highest number of articles, representing 37% of the total number of articles included in this review. This is followed by Applied Energy with a percentage of 25%. Moreover, 74 articles were published about the electricity system in Saudi Arabia, representing 36%, followed by the UAE with 18%.

The next section presents the descriptive statistics, including the analytical tools used, yearly publication, number of authors, keywords used, and page numbers of articles.

### 2.2 An overview of GCC electricity literature

#### 2.2.1 Overview of analytical tools used

As mentioned earlier, Connolly et al. (2010) classified the computer tools used to analyze the integration of renewable energy into seven categories. These seven categories are:

1. Simulation tools: These tools simulate the operation of the energy system in question to supply a set of energy demands.
2. Scenario tools: These usually combine a series of years or period into a long-term scenario.
3. Equilibrium tools: These aim to explain the behavior of supply, demand, and prices in an economy or in part of an economy (general or partial) with several markets.
4. Top-down tools: These are macroeconomic tools using general macroeconomic data to determine growth in energy prices and demands.
5. Bottom-up tools: These tools identify and analyze specific energy technologies and thereby identify investment options and alternatives.
6. Operation optimization tools: These tools optimize the operation of the energy system being studied.
7. Investment optimization tools: Lastly, these tools aim to optimize investments in an energy system.

The 206 articles considered in this literature review were classified using this categorization. Table 3 shows the categorization of the articles according to the analysis tools.

**Table 3:** Breakdown of published articles by analytical tool used.

No	Analytical Tool	Articles
1	Bottom-Up	Al-Marafie (1988); Al Baharna and Al Mahdi (1991); Nasser and El-Kalay (1991); Alawaji et al., (1995); Alnaser (1999); Akbaba (1999); Alajlan (1999); Al Suleimani and Nair (2000); Al Suleimani and Rao (2000); Maheshwari et al., (2001); Alnatheer (2006); Rehman et al., (2007); Kazim (2007); Darwish et al., (2008); Jowder (2009); Al-Badi et al., 2009a, Al-Badi et al., 2009b; Malik and Al-Badi (2009); Albadi et al., (2009); Darwish et al., (2010); Charabi and Gastli (2010); Gastli et al., (2010); Gastli and Charabi (2010); Sultan et al., (2010); Reiche (2010); Siddiqi and Anadon (2011); Alnaser and Alnaser (2011); Alotaibi (2011); Kazem (2011); Al-Badi et al. (2011a); Redha et al., (2011); Al-Badi and Albadi (2012); Rehman and El-Amin (2012); Rehman and Sahin (2012); Rahman and Khondaker (2012); Rahman et al., (2012); Al-Alili et al. (2012b); Popli et al., (2012); Mezher et al., (2012); Malik and Bouzguenda (2013); Abdul-Majeed et al., (2013); Hussein et al., (2013); Al-Amir and Abu-Hijleh (2013); Mokri et al., (2013); Chandarasekharam and Aref (2014); Ghaffour et al., (2014); Lashin and Al Arifi (2014); Abdmouleh et al., (2015); Baseer et al., (2015); Mondal et al., (2016); Ouda et al., (2016); Paleologos et al., (2016); Khondaker et al., (2016); Kumar et al., (2016); Jamil et al., (2016); Juaidi et al. (2016a); Gherboudj and Ghedira (2016); Mohan et al., (2016); Al-Maamary et al., 2017a, Al-Maamary et al., 2017b; Bou-Rabee et al., (2017); Nizami et al., (2017); Khan et al., (2017); Ramli et al., (2017); Mokheimer et al., (2017); Kouta et al., (2017); Alnaser (2018); Alnaser et al., (2018); Alsayegh et al., (2018); Almarshoud and Adam (2018)
2	Equilibrium	Ayyash et al., (1983); Ayyash (1983); Ayyash and Hammoudeh (1985); Al-Marafie et al., (1989); Kellow (1989); Al-Hinai et al., (1993); Burney and Al-Matrouk (1996); Dincer et al., (2004); Dincer et al., (2005); Eissa (2011); BuShehri and Wohlgenant (2012); Ahmad and Ramana (2014); Mondal et al., (2014); Matar et al., (2015); Juaidi et al. (2016b); Groissböck and Pickl (2016); Alasseri et al., (2017); Matar and Anwer (2017); Matar et al., (2017)
3	Investment Optimization	Alnatheer (2005); Al-Muhawesh and Qamber (2008); Farnoosh et al., (2014); Jayaraman et al., (2015); Almansoori and Betancourt-Torcat (2015); Jayaraman et al., (2017); Alshammari and Sarathy (2017); Baseer et al., (2017); Al Garni and Awasthi (2017); Parkinson et al., (2018)
4	Operational Optimization	Abdel-Aal and Al-Garni (1997); Badri et al., (1997); Ramanathan (2005); Al-Iriani (2005); Malik and Al-Zubeidi (2006); Malik (2007); Al-Sanea and Zedan (2008); AlRashidi and El-Naggar (2010); Al-Sanea and Zedan (2011); Shams et al., (2016); Atif and Al-Sulaiman (2017); Saghafifar and Gadalla (2017);
5	Scenario	Alnaser (1995); Al-Ismaily and Probert (1996); Al-Ismaily and Probert (1997); Al-Ajlan et al., (2006); Wood and Alsayegh (2014); Asif (2016); Treyer and Bauer (2016); Sgouridis et al., (2016); Matar (2018);
6	Simulation	Gari et al., (1988); Abdelrahman et al., (1993); Maheshwari and Al-Murad (2001); Alawaji (2001); Omar and Al-Ragom (2002); Radhi et al., (2009); Radhi (2009); Shaahid and El-Amin (2009); Rehman and Al-Hadhrami (2010); Radhi (2011); Krarti and Hajiah (2011); Gastli and Charabi (2011); Charabi et al., (2011); Taleb and Sharples (2011); Al-Sanea et al., (2012); Radhi (2012); Al-Masri and Abu-Hijleh (2012); Al-Alili et al. (2012a); AlFarra and Abu-Hijleh (2012); El Fadel et al., (2013); Shaahid et al., (2013); Shaahid et al., (2014); Aldossary et al., (2014); Rohani and Nour (2014); Krarti (2015); Al-Yahyai and Charabi (2015); Kharseh et al., (2015); Alrashed and Asif (2015); Mokheimer et al., (2015); Saghafifar and Gadalla (2015); AlAjmi et al., (2016); Al Busaidi et al., (2016); Mujeebu et al., (2016); Almarshoud (2016); Kaddoura et al., (2016); Al-Ugla et al., (2016); Abd-ur-Rehman and Al-Sulaiman (2016); Azar et al., (2016); De Wolf et al., (2017); Martín-Pomares et al., (2017); Rashwan et al., (2017); Rehman et al., (2017); Krarti et al., (2017); Kassem et al., (2017); Al-Sharafi et al., (2017); Rehman (2017); Gelan, 2018a, Gelan, 2018b; Krarti and Dubey (2018);
7	Top-Down	Al-Garni et al., (1994); Al-Ismaily and Probert (1995); Nizami and Al-Garni (1995); Abdel-Aal et al. (1997); Al-Faris (2002); Ben-Nakhi and Mahmoud (2002); Al-Iriani (2006); Squalli (2007); Rehman and Mohandes (2008); El-Sebaii et al., (2009); Mahmoud and Alajmi (2010); Ozturk and Acaravci (2011); Al-Badi et al. (2011b); Radhi and Sharples (2013); Al-Mulali and Tang (2013); Alkhathlan and Javid (2013); Mansouri et al., (2013); Al-Mulali and Ozturk (2014); Salahuddin and Gow (2014); Salahuddin et al. (2015); Jammazi and Aloui (2015); Alshehry and Belloumi (2015); Boräng et al.,2016; Sweidan and Alwaked (2016); Atalla and Hunt (2016); Osman et al., (2016); Hussain and Al-Alili (2016); Ghazal et al., (2016); Charfeddine and Khediri (2016); Bekhet et al., (2017); Hasanov et al., (2017); Mezghani and Haddad (2017); Mahalik et al., (2017); Hussain and AlAlili (2017); Azar and Al Ansari (2017); Salahuddin et al., (2018); Charfeddine et al., (2018)

#### 2.2.2 Temporal analysis of published research

**Fig. 1:**
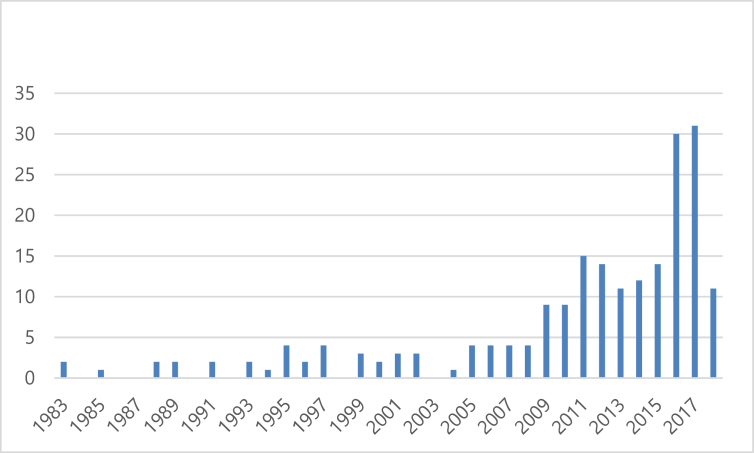
Distribution of electricity articles from 1983 to 2018.

Fig. 1 shows the increase in the number of articles on GCC electricity systems published from 1983 to 2018. There has clearly been an increase in the number of publications on the topic in recent years. There are three periods in the study period. The first period includes the years from 1983 to 2008. During this period, the minimum number of articles per year is zero and the maximum is four, with an average of approximately two articles per year. The second period goes from 2009 to 2015. The average number of articles is about 12, with a minimum of nine articles and a maximum of 15 articles per year. The third period is from 2016 to 2018. This period has a large number of publications, averaging 24 articles per year. The minimum is 11 articles, and the maximum is 31 articles published in 2017.

The increasing trend in the number of published articles concerning electricity in GCC countries matches the increasing trend of scientific publications in other fields. Bornmann and Mutz (2015) reviewed scientific publications from 1980 to 2012 using Web of Science (WoS) databases. They found that the global number of scientific publications experienced exponential growth, with an average annual increase of approximately 3%. Similarly, Sa'ed et al. (2015) analyzed the research output of 22 Arab countries published in 22 international Integrative and Complementary Medicine (ICM) journals indexed in the WoS databases. They identified 591 publications in 19 of these journals. Their analysis also showed an increase in the number of annual publications. In the period 1980–1989 (10 years), there were 29 publications. The number increased to 82 publications in the period 1990–1999. From the year 2000, the number of publications were presented on a yearly basis. In 2000, there were 25 publications. This number increased annually and reached 66 publications in 2013. In another study, Tadmouri and Tadmouri (2002) analyzed biomedical research in the Kingdom of Saudi Arabia during the period 1982–2000. They used the Science Citation Index (SCI) and PubMed databases and found 5,962 articles. The first article was published in 1982 and it was the only publication in that year. The number of publications increased annually, reaching 508 in the year 2000.

The general increase of scientific publications can be attributed to three reasons. The first reason is the global increase in the number of scientists. The second is the increasing number of scientific discoveries worth communicating to peers and the public. The third is the administrative pressure of academic institutions on their members to publish (Pautasso, 2012). These three reasons can also be applied to the increase in studies in GCC countries, which have experienced marked economic and academic growth in the last four decades. One more plausible reason for the increase in scientific publication on electricity in GCC countries could be the improvement in means of communication, such as the use of the Internet to share scientific publications with peers all over the world.

#### 2.2.3 Author statistics

**Fig. 2:**
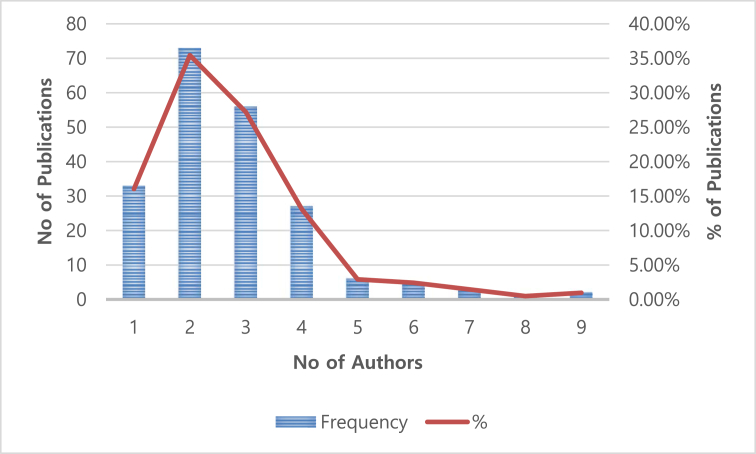
Distribution of electricity-related articles by number of authors (1983–2018).

Fig. 2 shows the frequency and percentage of authors of the articles considered in this study. A total of 33 articles (16.02%) were published by a single author, while two articles (0.97%) were published by nine authors, the maximum number of authors identified in this study. The average number of authors per article is 2.73. The highest number of articles (73) were published by two authors, which is the mode.

#### 2.2.4 Keywords statistics

Most of the articles surveyed included keywords. Table 4 shows the top 50 keywords used in these articles. “Renewable energy” is the most-used keyword in the articles. This reflects the importance of considering renewable energy to supply electricity in GCC countries. The second most-used keyword is “Saudi Arabia,” which appeared in 22 articles, and the third is “United Arab Emirates,” followed by “GCC” and “Energy consumption.”

**Table 4:** The 50 most-used keywords in GCC electricity articles.

No	Keywords	Number of Articles
1	Renewable energy	32
2	Saudi Arabia, Saudi Arabia (KSA), Kingdom of Saudi Arabia	22
3	United Arab Emirates (UAE), United Arab Emirates, UAE	19
4	GCC, GCC countries, Gulf Cooperation Council, Gulf Corporation Countries	17
5	Energy consumption	16
6	Carbon dioxide emission, Carbon emission, Carbon emissions, CO2 emission, CO2 emissions	14
7	Solar electricity generation, Solar electricity, Solar energy, Solar Power	14
8	Oman	13
9	Economic growth, Economics growth	11
10	Energy conservation	10
11	Solar radiation	10
12	Wind energy, Wind power	9
13	Solar	9
14	Photovoltaic, PV	8
15	Demand management, Demand side management, DSM	7
16	Wind	7
17	Kuwait	6
18	Electric energy consumption, Electricity consumption	6
19	Buildings, Existing buildings	5
20	Geographic information system, GIS	5
21	Energy efficiency	5
22	Electricity generation	5
23	Electricity, Electric power	5
24	Wind speeds, Wind speed	5
25	Waste-to-energy (WTE), Waste-to-energy	4
26	Solar irradiance	4
27	CSP	4
28	Capacity factor	4
29	Energy	4
30	Environment	4
31	Financial development	4
32	GHG emissions, Greenhouse gas emissions	4
33	Multi-criteria analysis, Multi-criteria decision, Multi-criteria decision analysis	4
34	Subsidy, Subsidies	4
35	Greenhouse gases, Greenhouse gases, Greenhouse gas	4
36	Duqm	3
37	Diesel generators, Diesel-engine	3
38	Demand response	3
39	BiPV	3
40	Battery	3
41	Artificial neural networks, Artificial neural network	3
42	Abu Dhabi	3
43	Energy policies, Energy policy	3
44	Energy savings, Energy saving	3
45	Neural networks	3
46	Nuclear energy, Nuclear power	3
47	Optimization	3
48	Solar photovoltaic	3
49	TRNSYS	3
50	Wind turbine	3

#### 2.2.5 Statistics based on length of publications

**Fig. 3:**
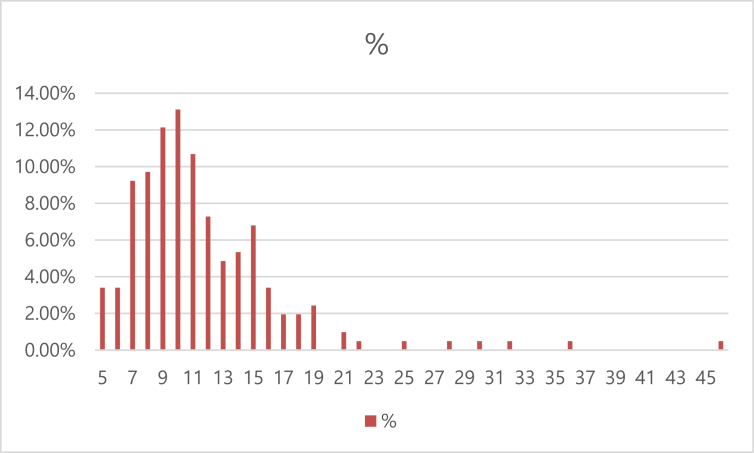
Distribution of electricity-related articles by number of pages (1983–2018).

Altogether, more than 2,300 pages have been published on GCC electricity issues in the five selected scientific journals. The number of pages per article ranges from five to 46 pages, with an average length of approximately 11.6 pages per article. Approximately 36% of the articles are between nine and 11 pages in length and about 79% of the articles are between 7 and 15 pages. Fig. 3 shows the distribution of the GCC electricity articles according to the number of pages.

### 2.3 Current and future research

To evaluate researchers’ current topics of interest concerning electricity in GCC countries, the keywords for the articles in the last three years (2016–2018) were analyzed. Table 5 shows the top five most used keywords in the surveyed articles. “Renewable energy” is the keyword that appears most often in the articles. This indicates that renewable energy is an emerging technology that GCC countries are seriously thinking of utilizing to provide electricity to their people. Moreover, “energy consumption” also appeared frequently, suggesting that high energy consumption puts pressure on GCC countries to meet the energy needs of the people.

**Table 5:** The five most-used keywords in GCC electricity articles in the period 2016–2018.

No	Keywords	Number of Articles
1	Renewable energy	13
2	Saudi Arabia, Kingdom of Saudi Arabia	10
3	GCC countries, Gulf Cooperation Council, Gulf Corporation Countries	10
4	United Arab Emirates (UAE), United Arab Emirates, UAE	9
5	Energy consumption	9

## 3 Conclusion

This study conducted a systematic analysis of current published research on electricity supply and demand in GCC countries during the period 1983–2018. The scope of the review was limited to the following five well-known energy journals: Applied Energy, Energy, Energy Economics, Energy Policy, and Renewable and Sustainable Energy Reviews. First, the study showed that the number of publications were very small at the beginning of the period. However, the number increased from 2009 and in the last three years (2016–2018), there was an average number of publications of approximately 24 per year. Second, more that 63% of the articles were published by two or three researchers, indicating a trend toward collaborated research. Third, “renewable energy” and “energy consumption” were among the highest number of most-used keywords in these studies, indicating the importance of renewable energy resources and controlling energy consumption in GCC countries.

This paper contributes to electricity literature in two distinctive ways. First, it shows how different electricity demand and supply issues in GCC countries are analyzed using the seven quantitative analytical tools classified by Connolly et al. (2010). The study found that the two most-used analytical tools are the bottom-up and simulation tools. The use of quantitative tools to analyze demand and supply is crucial because of the expensive nature of the electricity infrastructure and the need to analyze different scenarios by changing parameters. The second scientific contribution of this study is that it provides a comprehensive list of articles that addressed several issues in the electricity sector in GCC countries. This list can assist scientists and researchers in conducting further research. For example, a researcher may investigate the link between electricity consumption and economic growth in a specific country or in the GCC countries as a group. According to the literature, there are four hypotheses that can be tested concerning the nexus between electricity and economic growth: conservation, growth, feedback, and neutrality (Payne, 2010). Another possible area of research is the provision of demand forecasts for electricity consumption in GCC countries by considering the latest actions of energy conservation policies. Additionally, GCC countries are seriously thinking of using renewable resources such as solar electricity to provide energy to meet its growing demand. This study provides a list of the articles that researchers need to conduct their research in the above-mentioned areas as well as other possible research areas.

## Declarations

### Author contribution statement

Mohammed A. AlKhars: Conceived and designed the experiments; Performed the experiments; Analyzed and interpreted the data; Contributed reagents, materials, analysis tools or data; Wrote the paper.

### Funding statement

This research did not receive any specific grant from funding agencies in the public, commercial, or not-for-profit sectors.

### Competing interest statement

The authors declare no conflict of interest.

### Additional information

No additional information is available for this paper.
